# The molecular mechanisms driving *Plasmodium* cell division

**DOI:** 10.1042/BST20230403

**Published:** 2024-04-02

**Authors:** David S. Guttery, Mohammad Zeeshan, Anthony A. Holder, Rita Tewari

**Affiliations:** 1 School of Life Sciences, Queen's Medical Centre, University of Nottingham, Nottingham, U.K.; 2 Department of Genetics and Genome Biology, College of Life Sciences, University of Leicester, Leicester, U.K.; 3 Malaria Parasitology Laboratory, The Francis Crick Institute, London, U.K.

**Keywords:** cell division, closed mitosis, crystalloids, DNA replication, *Plasmodium*, reversible protein phosphorylation

## Abstract

Malaria, a vector borne disease, is a major global health and socioeconomic problem caused by the apicomplexan protozoan parasite *Plasmodium*. The parasite alternates between mosquito vector and vertebrate host, with meiosis in the mosquito and proliferative mitotic cell division in both hosts. In the canonical eukaryotic model, cell division is either by open or closed mitosis and karyokinesis is followed by cytokinesis; whereas in *Plasmodium* closed mitosis is not directly accompanied by concomitant cell division. Key molecular players and regulatory mechanisms of this process have been identified, but the pivotal role of certain protein complexes and the post-translational modifications that modulate their actions are still to be deciphered. Here, we discuss recent evidence for the function of known proteins in *Plasmodium* cell division and processes that are potential novel targets for therapeutic intervention. We also identify key questions to open new and exciting research to understand divergent *Plasmodium* cell division.

## Introduction

Cell division occurs through a tightly co-ordinated set of processes. Studies in several model eukaryotes including humans and yeast have highlighted highly conserved mechanisms of cell division [1]; however, variation in this process occurs, for example in the divergent branch of eukaryotes that includes the unicellular apicomplexan parasites. This group includes pathogens of socioeconomic importance such as *Toxoplasma* and *Plasmodium*, the causative agents of toxoplasmosis and malaria, respectively. These organisms have some conserved aspects of cellular development [2], but metabolic regulatory pathways, membrane biogenesis and cytoskeletal dynamics are highly divergent and subject to continuous intense research [3–7]. *Plasmodium* is one of the most devastating global causes of vector-borne disease, killing over 600 000 people per year [8]. In this review, we discuss recent developments in our understanding of *Plasmodium* cell division, focusing on closed mitosis during proliferation, development and transmission throughout the life-cycle in both mammalian host and mosquito vector.

### The malaria parasite life-cycle — morphologically distinct cells using diverse forms of cell division

**Figure 1.**
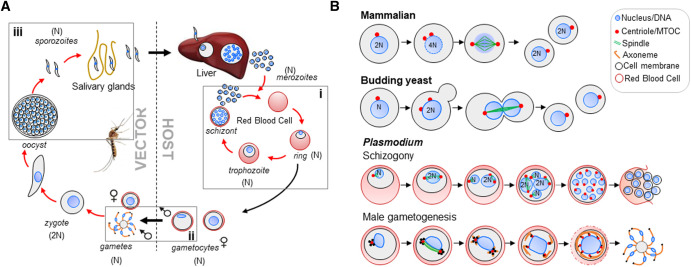
Plasmodium cell division and the malaria parasite life-cycle. (**A**) Life cycle of *Plasmodium* spp. highlighting schizogony in red blood cells (i), and male gametogenesis (ii) and sporogony (iii) in the mosquito midgut. (**B**) Modes of asexual cell division in *Plasmodium* (endomitosis) compared with model organisms (binary fission). In mammalian and yeast systems, open mitosis results in dissolution of the nuclear membrane at the G2/M transition and reassembly post-DNA segregation, with concomitant cytokinesis resulting in two individual daughter cells while in budding yeast the nuclear membrane remains intact and the chromosomes are separated on a spindle assembled within the nucleus. In *Plasmodium*, DNA replication is closed with replication of the genome and DNA segregation occurring within a single nucleus, with karyokinesis and cytokinesis at the end of this process resulting in eight haploid flagellate male gametes.

The *Plasmodium* life cycle is characterised by a series of morphologically distinct stages. Infection in mammals is initiated by injection of motile haploid sporozoites into the dermis during a mosquito blood-meal. Sporozoites eventually invade host hepatocytes and begin a proliferative stage in which there is DNA replication and karyokinesis (nuclear division) but no cytokinesis (cell division) until the end of the intracellular stage — exoerythrocytic schizogony. This produces thousands of haploid merozoites, which egress from the host cell and invade erythrocytes to begin cyclic asexual proliferation by intra-erythrocytic schizogony (Figure 1A(i)) and invasion of further erythrocytes. Schizogony produces a multinucleate cell (a coenocyte) and subsequent cytokinesis forms new merozoites. During this replicative phase, some parasites differentiate into male and female gametocytes that are arrested at the G0 phase of the cell cycle and are responsible for initiation of the sexual stage and transmission to the mosquito. Ingestion in the blood meal by a mosquito activates the gametocytes, with male(micro) gametocytes undergoing three rounds of rapid DNA replication and mitosis without karyokinesis (termed endomitosis) within 15 min, followed by karyokinesis and cytokinesis leading to the budding of eight haploid gametes. These motile gametes find and fertilise the mature female(macro) gamete (Figure 1A(ii)) to produce a diploid zygote [7], which over a period of 24 h elongates and differentiates into a crescent (or ‘banana’)-shaped motile ookinete, accompanied by DNA replication (to 4N) and the first stages of meiosis. The ookinete traverses the mosquito midgut wall and develops into an oocyst over a period of up to 21 days. Within the oocyst, sporogony proceeds with multiple rounds of DNA replication and karyokinesis, similar to schizogony, followed by cytokinesis to produce hundreds of motile haploid sporozoites (Figure 1A(iii)) that migrate to the mosquito salivary glands for transmission during the next bloodmeal.

### Schizogony in the mammalian host — a closed form of cell division

In the classical model system of mammalian cell division, mitosis is open with dissolution of the nuclear membrane. The replicated chromosomes condense, and the duplicated sister chromatids are separated on a microtubule-based bipolar spindle. In closed mitosis as found in budding yeast the nuclear membrane remains intact and the chromosomes are separated on a spindle assembled within the nucleus [9] (Figure 1B). In both cases karyokinesis and cytokinesis produce two daughter cells.

Mitotic cell division during *Plasmodium* schizogony is characterised by several rounds of DNA replication and asynchronous nuclear division (a process defined by DNA replication in the absence of cell division [10]), to produce a multinucleated single cell or coenocyte, and termed a schizont (reviewed extensively in [11,12]) (Figure 1B), followed by one round of cytokinesis at the end of schizogony to form merozoites. In both exo- and intra-erythrocytic schizogony mitosis is accompanied by rapid development and replication of organelles including the mitochondrion, the apicoplast (a plastid found in the Apicomplexa [13]), the inner-membrane complex (IMC), the basal membrane and organelles involved in host cell invasion such as the micronemes and rhoptries. The main difference between liver- and blood stage schizogony is that tens of thousands of merozoites are produced in hepatocytes [14]; whereas blood stage schizogony produces 8–32 merozoites each cycle [11] (Figure 1B).

In schizogony, nuclear and cell division differ from the canonical process. The model of eukaryotic cell cycle progression is of tightly regulated phases: a growth or gap phase (G1), DNA synthesis (S-phase), another gap phase preparing for mitosis (G2), and nuclear division and segmentation (mitosis — M phase). Mitosis is followed by cytokinesis when organelles and the cytoplasm are physically distributed between the two new daughter cells. In *Plasmodium,* following host cell invasion, the initial haploid ring and trophozoite stages resemble G1 phase cells, then the trophozoite enters schizogony characterised by multiple rounds of asynchronous DNA synthesis and nuclear division (S-phase) to produce multiple nuclei without immediate cytokinesis after each round [11,12]. Hence, schizogony appears to lack a G2 phase due its continual and cyclical use of S-phase [15]. The divergent nature of *Plasmodium* cell cycle progression is exemplified by a lack of canonical cell cycle checkpoint proteins including p53, ataxia telangiectasia and Rad3-related (ATR), ataxia telangiectasia mutated (ATM) and retinoblastoma protein (Rb) among others [15,16]. In addition, *Plasmodium* (and other Apicomplexa) has an atypical repertoire of cyclins, cyclin-dependent kinases (CDKs) and CDK-related kinases (CRKs). Orthologues of three cyclins (Cyc1, Cyc3 and Cyc4 [17]), and seven CDK or CDK-related kinases, protein kinase (PK) 5, PK6, the serine/threonine kinase MRK1, CRK1, CRK3, CDK4 and CRK5 have been identified [16]. However, while CRK5 is likely not essential for *Plasmodium* blood stage development, it has been shown to be key for male gametogenesis and interacts with the *Plasmodium-*specific cyclin SOC2 [18].

**Figure 2.**
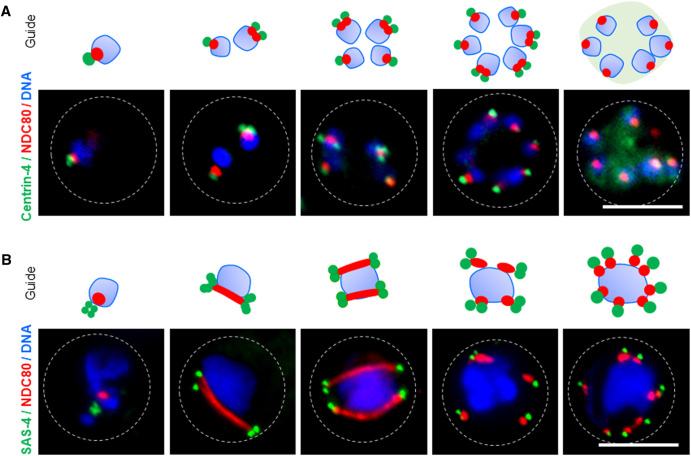
Live-cell imaging of cell division markers during two stages of proliferation in *Plasmodium*. (**A**) Live cell imaging using NDC80-mCherry (kinetochore marker) and Centrin-4-GFP (MTOC marker) showing spatio-temporal dynamics of the kinetochore and MTOC during blood stage schizogony. (**B**) Live cell imaging using NDC80-mCherry (kinetochore marker) and SAS4-GFP (MTOC/basal body marker) showing spatio-temporal dynamics of the kinetochore and basal body during male gametogenesis. Scale bar = 5 µm.

Studies using ultrastructural expansion microscopy (U-ExM) and Stimulated Emission Depletion Microscopy (STED) have generated an atlas of the three-dimensional structures and organelles that drive *Plasmodium* schizogony and their temporal profiles [19–21] (for extensive review on use of U-ExM to analyse *Plasmodium*, see [22]). The first cellular feature of schizogony is production of the hemispindle, defined as an array of microtubules that radiate into the nucleus from a single microtubule organising centre (MTOC) embedded in the nuclear membrane, and also referred to as the centriolar plaque [15,23]. Once DNA replication is complete, driven by phosphorylation of origin of replication (ORC) and minichromosome maintenance (MCM) complexes by cyclin-dependent kinases such as cdc2-related kinase 4 (CRK4) [24–26], the MTOC organises the microtubules into a mitotic spindle that segregates the replicated chromosomes. The centriolar plaque in *Plasmodium* asexual stages is a protein-dense structure devoid of chromatin and centrioles, with an intra- and extranuclear compartment [12,23]. Here, centrin-4 is located at the MTOC (Figure 2A) and forms a complex with centrin-1 and -3, but it is not essential for asexual stage development and proliferation, suggesting redundancy with, and possible replacement by, other centrins [27]. In addition, phosphorylation of centrins and an Sfi1-like centrin-interacting centriolar plaque protein (PfSlp — which has a key role in *Plasmodium* nuclear microtubule homeostasis [28]) may play an important role in kinetochore assembly and centrin localisation during schizogony. For example, in human cells, phosphorylation of centrins 1 and 2 by casein kinase 2 (CK2) regulates their binding to Sfi1 [29], and phosphorylation of Sfi1 by CDK1 is essential for yeast spindle pole body separation in G1/S phase [30]. The *Plasmodium* homologue of CDK1 is PK5 but is not essential at any stage of the life-cycle [31]; whereas the catalytic subunit of PfCK2 (PfCK2α) may play a role during late gametocytogenesis [32]. Finally, protein dephosphorylation is also important, with depletion of the protein phosphatase (PP) 1 that is located at kinetochores resulting in decreased DNA replication in *Plasmodium falciparum* asexual blood stages [33].

Throughout schizogony, several organelles develop in preparation for daughter cell (merozoite) formation. For example, biogenesis of apical organelles including the rhoptries and micronemes occurs *de novo*. The IMC, a double layered membranous structure located beneath the plasma membrane (PM) [16] is pulled around the newly-forming daughter cells by the basal complex (BC), whose integrity is maintained by the PPP-type pseudophosphatase PPP8 [34] and acts as a contractile ring to constrain and define daughter cell boundaries [35]. Several proteins are known to be essential for IMC and BC biogenesis, including IMC sub-compartment proteins (ISP)1 and 3, merozoite organising protein and CINCH [36–38], and others [39]. Transition from a multinucleated schizont to multiple, individual merozoites containing a single haploid nucleus occurs via PM invagination, redistribution of the BC to the PM and reorganisation of the IMC [40]. The PM and IMC subsequently fully enclose the newly forming merozoites, which are ultimately pinched off following the final segmentation of the genome and cytokinesis facilitated by schizont egress antigen-1 [41] (see also [12] for essential players in DNA segmentation). In addition, throughout schizogony the mitochondrion and apicoplast expand and are divided such that one of each is distributed to every merozoite [42,43]. Key components of this process include the dynamins DYN1 and DYN2 and autophagy-related protein 8 (for in depth review, see [11]).

### Male gametogenesis — a rapid, complex and highly co-ordinated endomitosis

Rapid cell division occurs in male gametocytes in the mosquito midgut. In the mosquito's blood meal, a temperature drop to ∼20–25°C, a rise in pH and the presence of the metabolic intermediate xanthurenic acid (XA) activate gametocytes to begin gametogenesis [44]. Three rounds of rapid, closed mitosis occur in the male gametocyte over 8–12 min, increasing DNA content from 1N to 8N, followed by karyokinesis, cytokinesis and the budding of eight microgametes in a process termed exflagellation (Figure 1B). In *P. falciparum* the falciform (or sickle-shaped) microgametocyte rounds-up upon activation, accompanied by formation of a single MTOC that develops into a tetrad of basal bodies attached to the spindle pole. Subsequent formation of eight basal bodies results in nucleation of eight axonemes, which elongate and segregate with each endomitotic division [44]. Each MTOC is closely aligned with a nuclear pore, and at exflagellation each newly formed gamete contains a single haploid genome in a nucleus that has been pulled through the basal body wrapped around an axoneme [45].

Male gametogenesis is complex and highly co-ordinated. Key phosphoregulators and other well-established components have been reviewed extensively elsewhere [44,46], with calcium-dependent PK 4 (CDPK4), serine-arginine PK 1 (SRPK1) and mitogen-activated PK 2 (MAPK2), as well as the metallo-dependent PP, PPM1, known to be essential drivers in *Plasmodium berghei* (Pb) [31,47,48]. The essential roles of CDPK4, SRPK1 and MAPK2 for *P. falciparum* male gametogenesis have been confirmed [49–51]; but an essential role for PfPPM1 is yet to be determined. Global phosphoproteome and interactome studies have begun to identify CDPK4, SRPK1 and MAPK2 substrates [47,52]. CDPK4 has three key roles during male gametogenesis mediated by three substrates: SOC1, SOC2 and SOC3. Interaction of CDPK4 with SOC1 within the first 10 s of activation facilitates the loading of the MCM2-7/Cdt1 complex onto the ORC1-5/Cdc6 complex, thereby assembling the pre-replicative complex and initiating DNA replication [52]. In the next 30 s, CDPK4 phosphorylation of SOC2, a microtubule-associated protein facilitates elongation of tubulin. Finally, 9 min after activation CDPK4 phosphorylates SOC3 to initiate axoneme motility and complete cytokinesis [52]. Additionally, a divergent cyclin/cyclin-dependent kinase (CRK5) was recently proposed to be in a signalling cascade with CDPK4 and SOC2 that drives origin of replication firing in *P. berghei* [18] and *P. falciparum* [53]. Deletion of SRPK1 results in a disruption of phosphosites that do not overlap with those of CDPK4 deletion mutants such as proteins involved in microtubule motor activity, actin binding and origin of replication recognition, suggesting that SRPK1 has different substrates to CDPK4 in addition to being regulated itself by CDPK4 [47]. However, some altered phosphosites were common to both deletion mutants, including those on proteins involved in microtubule-based movement and ATP binding. In *P. falciparum*, SRPK1 gene deletion using CRISPR/Cas9 technology affected transcript splicing and abundance for genes coding many proteins involved in microtubule morphogenesis, cyclic nucleotide metabolism, lipid metabolism, osmophilic body formation and crystalloid components [49]. The substrates of MAPK2 are less known; however, PbMAPK2 is essential for chromosome condensation prior to exflagellation [54], potentially driving axoneme motility, and in *P. falciparum* it has been shown to co-ordinate axoneme beating [51].

Recently, new components that may have vital functions in male gametogenesis and exflagellation have been identified. Gametogenesis essential protein 1 (GEP1) has recently been identified as a master trigger for gametogenesis, essential for XA-stimulated activation. Disruption of GEP1 gene abolished XA-stimulated cyclic GMP synthesis and completely ablated downstream cellular and signalling events [55]. Deletion of a gene encoding a *P. falciparum* protein with an AT-rich DNA interaction domain (PfARID) resulted in down-regulation of genes important for gametogenesis, with complete ablation of exflagellation [56]. Other players include Pb22, APC3 and radial spoke protein 9 (RSP9) in *P. berghei* [57–59], p25α in *Plasmodium yoelii* [60] and the patatin-like phospholipase, PLP2 in *P. falciparum* [61]. For a complete overview of essential regulators of gametogenesis, see [44].

### Kinetochore and spindle dynamics driving male gametocyte cell division

The rapid rounds of mitosis during male gametogenesis are driven by formation of microtubular spindles to facilitate segregation of sister chromatids to the spindle poles. Within the first few seconds following microgametocyte activation, clusters of genes coding proteins involved in microtubule activity, microtubule binding and microtubule-based movement are activated [47], along with a tetrad of kinetosomes that form the MTOCs and kinetochores [62,63]. These proteins anchor mitotic spindles in the nucleoplasm and facilitate growth of axonemes in the cytoplasm [64]. A combination of live-cell imaging of tagged kinesin-8B, NDC80, kinesin-5 and spindle-assembly abnormal (SAS) 4 with transmission electron microscopy have revealed key aspects of the highly dynamic kinetosome and MTOC formation [62,63,65,66]. NDC80 is a highly conserved component of the kinetochore that mediates chromosome attachment to spindle microtubules and can be used as a marker for chromosome segregation [67], Following completion of each round of male gametocyte chromosome segregation, NDC80 is located at distinct foci in the nucleus, but extends to form a bridge across the entire nuclear body during segregation, while the basal body marker, SAS4 remains associated with the MTOC, making focal points in the cytoplasm [65] (Figure 2B). Kinesin-5, a molecular motor that is structurally and functionally conserved across eukaryotes and involved in spindle pole separation during mitosis [68], is also found located on mitotic spindles, colocalised with NDC80 during male gametogenesis. This interaction may be regulated by PP1, since PP1 co-localises with NDC80 at the kinetochores [69].

Four recent studies have highlighted the importance of microtubule end-binding proteins (EBs) for spindle-kinetochore attachment, chromosome segregation and partitioning of nuclei during male gametogenesis. EB1, found only in *Plasmodium*, has been found to decorate the full-length of spindle microtubules during male gametogenesis [64,70,71]. Deletion of the EB1 gene results in anucleate *P. falciparum* male gametes [72], and complete ablation of oocyst development in both *P. berghei* and *P. falciparum* [70–72]. Regulation of EB1 activity is co-ordinated by the divergent Aurora Kinase 2, which associates with microtubule proteins near the spindle-kinetochore interface, and potentially forms a microtubule-anchoring complex comprised of EB1, MISFIT and myosin K (MyoK) [71].

### Sporogony in the developing oocyst is similar to schizogony — the final rounds of cell division in the mosquito host

In the oocyst there is a lengthy period of growth over 1–3 weeks with several rounds of DNA replication and karyokinesis followed by a final step of cytokinesis to generate hundreds of haploid sporozoites in a process termed sporogony. The density of oocysts on the midgut wall is heavily determined by the gametocyte density in the bloodmeal [73] and, despite the lengthy process (especially when compared with male gametogenesis), oocyst development is highly dynamic.

Differentiation of the oocyst from the ookinete following attachment to the basal lamina of the midgut wall is thought to be triggered by interaction of P25/28, circumsporozoite- and TRAP-related protein and secreted ookinete adhesive protein with laminin [74]. This initiates sporogony with an increase in the size of the oocyst to a diameter of 50–60 µm, accompanied by retraction of the parasite PM around the developing sporoblast forming deep invaginations and cytoplasmic lobes. Ultrastructural microscopy and live-cell imaging have so far not identified when cytokinesis occurs during this process, although the kinetochore marker NDC80 is revealed at multiple foci adjacent to the nuclear DNA [63]. Several proteins are known to be essential for completion of sporogony, for de novo fatty acid synthesis (FAS) and for scavenging components from the host (in particular the FASII pathway [75]), DNA replication and metabolism [76] and evasion of the mosquito immune system [77]. Reverse genetic studies have shown that deletion of genes encoding 3-hydroxyacyl-CoA dehydratase (DEH — a component of FAS), the DNA repair protein meiotic recombination 11 (MRE11), P-type cyclin, cyclin 3 (CYC3) and several of the Limulus clotting factor C, Cochlin and Lgl1 (LCCL) lectin domain adhesive-like proteins (LAPs — essential for crystalloid biogenesis [78], see below) results in complete ablation of sporogony and parasite transmission [44,79]. Reversible protein phosphorylation also appears to facilitate sporogony, since deletion of the genes for PKs cyclin-G associated kinase (GAK) and PK7, along with the metallo-dependent PP, PPM5 also completely ablates sporogony [31,48]. Recent studies have identified further regulators of sporogony: *Plasmodium* Infection of the Mosquito Midgut Screen (PIMMS)-01, -57, and -22 [80], and aquaporin 2 [81].

An organelle that may be crucial for oocyst development and sporogony is the crystalloid. These tightly packed, small spherical structures are found only in ookinetes and young oocysts but their origin and function remained elusive until recently (reviewed extensively in [82]). Namely, deletion of the six LCCL LAPs results in failure of the oocyst to undergo sporogony [78,79,83,84], as do deletion mutants of the recently discovered CRystalloid Oocyst Not Evolving gene [85], among others [82].

### Open questions

1. The *Plasmodium* cell cycle lacks a G2 phase and canonical eukaryotic cell cycle checkpoint proteins are not encoded in the genome, whereas there is an unusual repertoire of cyclins/CRKs. What is the biological significance of this diversity, what cyclin/CRK complexes are formed and what do they regulate?
2. How does the parasite switch from multinucleated division to endomitosis?
3. How is the MTOC organised in schizogony, sporogony and male gametogenesis and how does it co-ordinate chromosome segregation, axoneme assembly and DNA segmentation?
4. How do hypnozoites (dormant liver-stage parasites [11,86]) halt schizogony and how are they reactivated?
5. How does the absence of chromosome condensation affect nuclear division?
6. What factors determine the different number of progeny cells produced at each stage of the parasite's life-cycle?

Answering these questions will require a combination of reverse genetics, state-of-the-art microscopy, cell biology, proteomics, phosphoproteomics and advanced bioinformatics, which will uncover exciting lines of research to shed light on the divergent aspects of *Plasmodium* cell division.

## Perspectives

- Asexual cell division in *Plasmodium* is divergent from that of canonical eukaryotic models and proceeds via several rounds of DNA replication and endomitotic division with or without karyokinesis and terminal cytokinesis.
- Numerous studies have elucidated essential roles for key molecular regulators of *Plasmodium* cell division in blood stages of the mammalian host and in the mosquito vector.
- A combination of cutting-edge cell, molecular, and biophysical techniques will be required to determine how and why *Plasmodium* cell division is divergent from that in model systems, and this knowledge may identify novel targets for therapeutic intervention.

## Open Access

Open access for this article was enabled by the participation of University of Nottingham in an all-inclusive *Read & Publish* agreement with Portland Press and the Biochemical Society under a transformative agreement with JISC.

## Abbreviations

BC: basal complex
CDPK4: calcium-dependent protein kinase 4
FAS: fatty acid synthesis
GEP1: gametogenesis essential protein 1
IMC: inner-membrane complex
LAP: lectin domain adhesive-like protein
MAPK2: mitogen-activated protein kinase 2
MTOC: microtubule organising centre
PK: protein kinase
PM: plasma membrane
PP: protein phosphatase
SRPK1: serine-arginine protein kinase 1
XA: xanthurenic acid

## References

[1] Harashima, H., Dissmeyer, N. and Schnittger, A. (2013) Cell cycle control across the eukaryotic kingdom. Trends Cell Biol. 23, 345–356 10.1016/j.tcb.2013.03.002 23566594

[2] White, M.W. and Suvorova, E.S. (2018) Apicomplexa cell cycles: something old, borrowed, lost, and new. Trends Parasitol. 34, 759–771 10.1016/j.pt.2018.07.006 30078701 PMC6157590

[3] Harding, C.R. and Frischknecht, F. (2020) The riveting cellular structures of apicomplexan parasites. Trends Parasitol. 36, 979–991 10.1016/j.pt.2020.09.001 33011071

[4] Shanmugasundram, A., Gonzalez-Galarza, F.F., Wastling, J.M., Vasieva, O. and Jones, A.R. (2013) Library of Apicomplexan Metabolic Pathways: a manually curated database for metabolic pathways of apicomplexan parasites. Nucleic Acids Res. 41, D706–D713 10.1093/nar/gks1139 23193253 PMC3531055

[5] Gubbels, M.J., Coppens, I., Zarringhalam, K., Duraisingh, M.T. and Engelberg, K. (2021) The modular circuitry of apicomplexan cell division plasticity. Front. Cell. Infect. Microbiol. 11, 670049 10.3389/fcimb.2021.670049 33912479 PMC8072463

[6] Beck, J.R. and Ho, C.M. (2021) Transport mechanisms at the malaria parasite-host cell interface. PLoS Pathog. 17, e1009394 10.1371/journal.ppat.1009394 33793667 PMC8016102

[7] Guttery, D.S., Zeeshan, M., Holder, A.A., Tromer, E.C. and Tewari, R. (2023) Meiosis in Plasmodium: how does it work? Trends Parasitol. 39, 812–821 10.1016/j.pt.2023.07.002 37541799

[8] Monroe, A., Williams, N.A., Ogoma, S., Karema, C. and Okumu, F. (2022) Reflections on the 2021 World Malaria Report and the future of malaria control. Malar. J. 21, 154 10.1186/s12936-022-04178-7 35624483 PMC9137259

[9] Gubbels, M.J., Keroack, C.D., Dangoudoubiyam, S., Worliczek, H.L., Paul, A.S., Bauwens, C. et al. (2020) Fussing about fission: defining variety among mainstream and exotic apicomplexan cell division modes. Front. Cell. Infect. Microbiol. 10, 269 10.3389/fcimb.2020.00269 32582569 PMC7289922

[10] Gandarillas, A., Molinuevo, R. and Sanz-Gomez, N. (2018) Mammalian endoreplication emerges to reveal a potential developmental timer. Cell Death Differ. 25, 471–476 10.1038/s41418-017-0040-0 29352263 PMC5864232

[11] Roques, M., Bindschedler, A., Beyeler, R. and Heussler, V.T. (2023) Same, same but different: exploring Plasmodium cell division during liver stage development. PLoS Pathog. 19, e1011210 10.1371/journal.ppat.1011210 36996035 PMC10062574

[12] Voss, Y., Klaus, S., Guizetti, J. and Ganter, M. (2023) Plasmodium schizogony, a chronology of the parasite's cell cycle in the blood stage. PLoS Pathog. 19, e1011157 10.1371/journal.ppat.1011157 36862652 PMC9980825

[13] Elaagip, A., Absalon, S. and Florentin, A. (2022) Apicoplast dynamics during Plasmodium cell cycle. Front. Cell. Infect. Microbiol. 12, 864819 10.3389/fcimb.2022.864819 35573785 PMC9100674

[14] Vaughan, A.M. and Kappe, S.H.I. (2017) Malaria parasite liver infection and exoerythrocytic biology. Cold Spring Harb. Perspect. Med. 7, a025486 10.1101/cshperspect.a025486 PMC5453383 28242785

[15] Gerald, N., Mahajan, B. and Kumar, S. (2011) Mitosis in the human malaria parasite *Plasmodium falciparum*. Eukaryot. Cell 10, 474–482 10.1128/EC.00314-10 21317311 PMC3127633

[16] Matthews, H., Duffy, C.W. and Merrick, C.J. (2018) Checks and balances? DNA replication and the cell cycle in *Plasmodium*. Parasit. Vectors 11, 216 10.1186/s13071-018-2800-1 29587837 PMC5872521

[17] Roques, M., Wall, R.J., Douglass, A.P., Ramaprasad, A., Ferguson, D.J., Kaindama, M.L. et al. (2015) Plasmodium P-type cyclin CYC3 modulates endomitotic growth during oocyst development in mosquitoes. PLoS Pathog. 11, e1005273 10.1371/journal.ppat.1005273 26565797 PMC4643991

[18] Balestra, A.C., Zeeshan, M., Rea, E., Pasquarello, C., Brusini, L., Mourier, T. et al. (2020) A divergent cyclin/cyclin-dependent kinase complex controls the atypical replication of a malaria parasite during gametogony and transmission. Elife 9, e56474 10.7554/eLife.56474 32568069 PMC7308089

[19] Liffner, B., Cepeda Diaz, A.K., Blauwkamp, J., Anaguano, D., Frolich, S., Muralidharan, V. et al. (2023) Atlas of *Plasmodium falciparum* intraerythrocytic development using expansion microscopy. bioRxiv 10.1101/2023.03.22.533773 PMC10727503 38108809

[20] Mehnert, A.K., Simon, C.S. and Guizetti, J. (2019) Immunofluorescence staining protocol for STED nanoscopy of Plasmodium-infected red blood cells. Mol. Biochem. Parasitol. 229, 47–52 10.1016/j.molbiopara.2019.02.007 30831155

[21] Liffner, B. and Absalon, S. (2021) Expansion microscopy reveals *Plasmodium falciparum* blood-stage parasites undergo anaphase with a chromatin bridge in the absence of mini-Chromosome maintenance complex binding protein. Microorganisms 9, 2306 10.3390/microorganisms9112306 PMC8620465 34835432

[22] Liffner, B. and Absalon, S. (2023) Expansion microscopy of apicomplexan parasites. Mol. Microbiol. 1–17 10.1111/mmi.15135 37571814

[23] Simon, C.S., Funaya, C., Bauer, J., Vobeta, Y., Machado, M., Penning, A. et al. (2021) An extended DNA-free intranuclear compartment organizes centrosome microtubules in malaria parasites. Life Sci. Alliance 4, e202101199 10.26508/lsa.202101199 34535568 PMC8473725

[24] Ganter, M., Goldberg, J.M., Dvorin, J.D., Paulo, J.A., King, J.G., Tripathi, A.K. et al. (2017) *Plasmodium falciparum* CRK4 directs continuous rounds of DNA replication during schizogony. Nat. Microbiol. 2, 17017 10.1038/nmicrobiol.2017.17 28211852 PMC5328244

[25] Machado, M., Klaus, S., Klaschka, D., Guizetti, J. and Ganter, M. (2023) Plasmodium falciparum CRK4 links early mitotic events to the onset of S-phase during schizogony. mBio 14, e0077923 10.1128/mbio.00779-23 37345936 PMC10470535

[26] Totanes, F.I.G., Gockel, J., Chapman, S.E., Bartfai, R., Boemo, M.A. and Merrick, C.J. (2023) A genome-wide map of DNA replication at single-molecule resolution in the malaria parasite *Plasmodium falciparum*. Nucleic Acids Res. 51, 2709–2724 10.1093/nar/gkad093 36808528 PMC10085703

[27] Roques, M., Stanway, R.R., Rea, E.I., Markus, R., Brady, D., Holder, A.A. et al. (2019) Plasmodium centrin PbCEN-4 localizes to the putative MTOC and is dispensable for malaria parasite proliferation. Biol. Open 8, bio036822 10.1242/bio.036822 30541825 PMC6361220

[28] Wenz, C., Simon, C.S., Romao, T.P., Sturmer, V.S., Machado, M., Klages, N. et al. (2023) An Sfi1-like centrin-interacting centriolar plaque protein affects nuclear microtubule homeostasis. PLoS Pathog. 19, e1011325 10.1371/journal.ppat.1011325 37130129 PMC10180636

[29] Grecu, D. and Assairi, L. (2014) CK2 phosphorylation of human centrins 1 and 2 regulates their binding to the DNA repair protein XPC, the centrosomal protein Sfi1 and the phototransduction protein transducin beta. FEBS Open Bio 4, 407–419 10.1016/j.fob.2014.04.002 PMC4050191 24918055

[30] Elserafy, M., Saric, M., Neuner, A., Lin, T.C., Zhang, W., Seybold, C. et al. (2014) Molecular mechanisms that restrict yeast centrosome duplication to one event per cell cycle. Curr. Biol. 24, 1456–1466 10.1016/j.cub.2014.05.032 24954044

[31] Tewari, R., Straschil, U., Bateman, A., Böhme, U., Cherevach, I., Gong, P. et al. (2010) The systematic functional analysis of Plasmodium protein kinases identifies essential regulators of mosquito transmission. Cell Host Microbe 8, 377–387 10.1016/j.chom.2010.09.006 20951971 PMC2977076

[32] Hitz, E., Gruninger, O., Passecker, A., Wyss, M., Scheurer, C., Wittlin, S. et al. (2021) The catalytic subunit of *Plasmodium falciparum* casein kinase 2 is essential for gametocytogenesis. Commun. Biol. 4, 336 10.1038/s42003-021-01873-0 33712726 PMC7954856

[33] Paul, A.S., Miliu, A., Paulo, J.A., Goldberg, J.M., Bonilla, A.M., Berry, L. et al. (2020) Co-option of *Plasmodium falciparum* PP1 for egress from host erythrocytes. Nat. Commun. 11, 3532 10.1038/s41467-020-17306-1 32669539 PMC7363832

[34] Morano, A.A., Rudlaff, R.M. and Dvorin, J.D. (2023) A PPP-type pseudophosphatase is required for the maintenance of basal complex integrity in *Plasmodium falciparum*. Nat. Commun. 14, 3916 10.1038/s41467-023-39435-z 37400439 PMC10317984

[35] Rudlaff, R.M., Kraemer, S., Streva, V.A. and Dvorin, J.D. (2019) An essential contractile ring protein controls cell division in *Plasmodium falciparum*. Nat. Commun. 10, 2181 10.1038/s41467-019-10214-z 31097714 PMC6522492

[36] Poulin, B., Patzewitz, E.M., Brady, D., Silvie, O., Wright, M.H., Ferguson, D.J. et al. (2013) Unique apicomplexan IMC sub-compartment proteins are early markers for apical polarity in the malaria parasite. Biol. Open 2, 1160–1170 10.1242/bio.20136163 24244852 PMC3828762

[37] Qian, P., Wang, X., Zhong, C.Q., Wang, J., Cai, M., Nguitragool, W. et al. (2022) Inner membrane complex proteomics reveals a palmitoylation regulation critical for intraerythrocytic development of malaria parasite. Elife 11, e77447 10.7554/eLife.77447 35775739 PMC9293000

[38] Absalon, S., Robbins, J.A. and Dvorin, J.D. (2016) An essential malaria protein defines the architecture of blood-stage and transmission-stage parasites. Nat. Commun. 7, 11449 10.1038/ncomms11449 27121004 PMC4853479

[39] Ferreira, J.L., Heincke, D., Wichers, J.S., Liffner, B., Wilson, D.W. and Gilberger, T.W. (2020) The dynamic roles of the inner membrane complex in the multiple stages of the malaria parasite. Front. Cell. Infect. Microbiol. 10, 611801 10.3389/fcimb.2020.611801 33489940 PMC7820811

[40] Rudlaff, R.M., Kraemer, S., Marshman, J. and Dvorin, J.D. (2020) Three-dimensional ultrastructure of *Plasmodium falciparum* throughout cytokinesis. PLoS Pathog. 16, e1008587 10.1371/journal.ppat.1008587 32511279 PMC7302870

[41] Perrin, A.J., Bisson, C., Faull, P.A., Renshaw, M.J., Lees, R.A., Fleck, R.A. et al. (2021) Malaria parasite schizont egress antigen-1 plays an essential role in nuclear segregation during schizogony. mBio 12, e03377-20 10.1128/mBio.03377-20 33688001 PMC8092294

[42] van Dooren, G.G., Marti, M., Tonkin, C.J., Stimmler, L.M., Cowman, A.F. and McFadden, G.I. (2005) Development of the endoplasmic reticulum, mitochondrion and apicoplast during the asexual life cycle of *Plasmodium falciparum*. Mol. Microbiol. 57, 405–419 10.1111/j.1365-2958.2005.04699.x 15978074

[43] Verhoef, J.M.J., Boshoven, C., Evers, F., Akkerman, L.J., Gijsbrechts, B.C.A., van de Vegte-Bolmer, M. et al. (2024) Detailing organelle division and segregation in *Plasmodium falciparum*. bioRxiv 10.1101/2024.01.30.577899 PMC11535888 39485315

[44] Guttery, D.S., Zeeshan, M., Ferguson, D.J.P., Holder, A.A. and Tewari, R. (2022) Division and transmission: malaria parasite development in the mosquito. Annu. Rev. Microbiol. 76, 113–134 10.1146/annurev-micro-041320-010046 35609946

[45] Rashpa, R. and Brochet, M. (2022) Expansion microscopy of Plasmodium gametocytes reveals the molecular architecture of a bipartite microtubule organisation centre coordinating mitosis with axoneme assembly. PLoS Pathog. 18, e1010223 10.1371/journal.ppat.1010223 35077503 PMC8789139

[46] Dash, M., Sachdeva, S., Bansal, A. and Sinha, A. (2022) Gametogenesis in Plasmodium: delving deeper to connect the dots. Front. Cell. Infect. Microbiol. 12, 877907 10.3389/fcimb.2022.877907 35782151 PMC9241518

[47] Invergo, B.M., Brochet, M., Yu, L., Choudhary, J., Beltrao, P. and Billker, O. (2017) Sub-minute phosphoregulation of cell cycle systems during Plasmodium gamete formation. Cell Rep. 21, 2017–2029 10.1016/j.celrep.2017.10.071 29141230 PMC5700370

[48] Guttery, D.S., Poulin, B., Ramaprasad, A., Wall, R.J., Ferguson, D.J., Brady, D. et al. (2014) Genome-wide functional analysis of Plasmodium protein phosphatases reveals key regulators of parasite development and differentiation. Cell Host Microbe 16, 128–140 10.1016/j.chom.2014.05.020 25011111 PMC4094981

[49] Kumar, S., Baranwal, V.K., Leeb, A.S., Haile, M.T., Oualim, K.M.Z., Hertoghs, N. et al. (2022) PfSRPK1 regulates asexual blood stage schizogony and is essential for male gamete formation. Microbiol. Spectr. 10, e0214122 10.1128/spectrum.02141-22 36094218 PMC9602455

[50] Kumar, S., Haile, M.T., Hoopmann, M.R., Tran, L.T., Michaels, S.A., Morrone, S.R. et al. (2021) *Plasmodium falciparum* calcium-dependent protein kinase 4 is critical for male gametogenesis and transmission to the mosquito vector. mBio 12, e0257521 10.1128/mBio.02575-21 34724830 PMC8561384

[51] Hitz, E., Balestra, A.C., Brochet, M. and Voss, T.S. (2020) PfMAP-2 is essential for male gametogenesis in the malaria parasite *Plasmodium falciparum*. Sci. Rep. 10, 11930 10.1038/s41598-020-68717-5 32681115 PMC7368081

[52] Fang, H., Klages, N., Baechler, B., Hillner, E., Yu, L., Pardo, M. et al. (2017) Multiple short windows of calcium-dependent protein kinase 4 activity coordinate distinct cell cycle events during Plasmodium gametogenesis. Elife 6, e26524 10.7554/eLife.26524 28481199 PMC5457135

[53] Kumar, S., Gargaro, O.R. and Kappe, S.H.I. (2022) *Plasmodium falciparum* CRK5 is critical for male gametogenesis and infection of the mosquito. mBio 13, e0222722 10.1128/mbio.02227-22 36154191 PMC9600428

[54] Guttery, D.S., Ferguson, D.J.P., Poulin, B., Xu, Z., Straschil, U., Klop, O. et al. (2012) A putative homologue of CDC20/CDH1 in the malaria parasite is essential for male gamete development. PLoS Pathog. 8, e1002554 10.1371/journal.ppat.1002554 22383885 PMC3285604

[55] Jiang, Y., Wei, J., Cui, H., Liu, C., Zhi, Y., Jiang, Z. et al. (2020) An intracellular membrane protein GEP1 regulates xanthurenic acid induced gametogenesis of malaria parasites. Nat. Commun. 11, 1764 10.1038/s41467-020-15479-3 32273496 PMC7145802

[56] Kumar, S., Baranwal, V.K., Haile, M.T., Oualim, K.M.Z., Abatiyow, B.A., Kennedy, S.Y. et al. (2022) PfARID regulates *P. falciparum* malaria parasite male gametogenesis and female fertility and is critical for parasite transmission to the mosquito vector. mBio 13, e0057822 10.1128/mbio.00578-22 35638735 PMC9239086

[57] Liu, F., Yang, F., Wang, Y., Hong, M., Zheng, W., Min, H. et al. (2021) A conserved malaria parasite antigen Pb22 plays a critical role in male gametogenesis in *Plasmodium berghei*. Cell Microbiol. 23, e13294 10.1111/cmi.13294 33222390 PMC8194029

[58] Wall, R.J., Ferguson, D.J.P., Freville, A., Franke-Fayard, B., Brady, D., Zeeshan, M. et al. (2018) Plasmodium APC3 mediates chromosome condensation and cytokinesis during atypical mitosis in male gametogenesis. Sci. Rep. 8, 5610 10.1038/s41598-018-23871-9 29618731 PMC5884774

[59] Ramakrishnan, C., Fort, C., Marques, S.R., Ferguson, D.J.P., Gransagne, M., Baum, J. et al. (2023) Radial spoke protein 9 is necessary for axoneme assembly in Plasmodium but not in trypanosomatid parasites. J. Cell Sci. 136, cs260655 10.1242/jcs.260655 PMC10309588 37288670

[60] Zhang, C., Li, D., Meng, Z., Zhou, J., Min, Z., Deng, S. et al. (2022) Pyp25α is required for male gametocyte exflagellation. Pathog. Dis. 80, ftac043 10.1093/femspd/ftac043 36316012

[61] Singh, P., Alaganan, A., More, K.R., Lorthiois, A., Thiberge, S., Gorgette, O. et al. (2019) Role of a patatin-like phospholipase in *Plasmodium falciparum* gametogenesis and malaria transmission. Proc. Natl Acad. Sci. U.S.A. 116, 17498–17508 10.1073/pnas.1900266116 31413195 PMC6717283

[62] Zeeshan, M., Ferguson, D.J., Abel, S., Burrrell, A., Rea, E., Brady, D. et al. (2019) Kinesin-8B controls basal body function and flagellum formation and is key to malaria transmission. Life Sci. Alliance 2, e201900488 10.26508/lsa.201900488 31409625 PMC6696982

[63] Zeeshan, M., Pandey, R., Ferguson, D.J.P., Tromer, E.C., Markus, R., Abel, S. et al. (2020) Real-time dynamics of Plasmodium NDC80 reveals unusual modes of chromosome segregation during parasite proliferation. J. Cell Sci. 134, jcs245753 10.1242/jcs.245753 32501284 PMC7340582

[64] Li, J., Shami, G.J., Cho, E., Liu, B., Hanssen, E., Dixon, M.W.A. et al. (2022) Repurposing the mitotic machinery to drive cellular elongation and chromatin reorganisation in *Plasmodium falciparum* gametocytes. Nat. Commun. 13, 5054 10.1038/s41467-022-32579-4 36030238 PMC9419145

[65] Zeeshan, M., Brady, D., Markus, R., Vaughan, S., Ferguson, D., Holder, A.A. et al. (2022) Plasmodium SAS4: basal body component of male cell which is dispensable for parasite transmission. Life Sci. Alliance 5, e202101329 10.26508/lsa.202101329 35550346 PMC9098390

[66] Zeeshan, M., Brady, D., Stanway, R.R., Moores, C.A., Holder, A.A. and Tewari, R. (2020) *Plasmodium berghei* kinesin-5 associates with the spindle apparatus during cell division and is important for efficient production of infectious sporozoites. Front. Cell. Infect. Microbiol. 10, 583812 10.3389/fcimb.2020.583812 33154955 PMC7591757

[67] Vader, G. and Musacchio, A. (2017) The greatest kinetochore show on earth. EMBO Rep. 18, 1473–1475 10.15252/embr.201744541 28720649 PMC5579379

[68] Mann, B.J. and Wadsworth, P. (2019) Kinesin-5 regulation and function in mitosis. Trends Cell Biol. 29, 66–79 10.1016/j.tcb.2018.08.004 30220581

[69] Zeeshan, M., Pandey, R., Subudhi, A.K., Ferguson, D.J.P., Kaur, G., Rashpa, R. et al. (2021) Protein phosphatase 1 regulates atypical mitotic and meiotic division in Plasmodium sexual stages. Commun. Biol. 4, 760 10.1038/s42003-021-02273-0 34145386 PMC8213788

[70] Yang, S., Cai, M., Huang, J., Zhang, S., Mo, X., Jiang, K. et al. (2023) EB1 decoration of microtubule lattice facilitates spindle-kinetochore lateral attachment in Plasmodium male gametogenesis. Nat. Commun. 14, 2864 10.1038/s41467-023-38516-3 37208365 PMC10199041

[71] Zeeshan, M., Rea, E., Abel, S., Vukusic, K., Markus, R., Brady, D. et al. (2023) Plasmodium ARK2 and EB1 drive unconventional spindle dynamics, during chromosome segregation in sexual transmission stages. Nat. Commun. 14, 5652 10.1038/s41467-023-41395-3 37704606 PMC10499817

[72] Mauer, S., Camargo, N., Abatiyow, B.A., Gargaro, O.R., Kappe, S.H.I. and Kumar, S. (2023) Plasmodium microtubule-binding protein EB1 is critical for partitioning of nuclei in male gametogenesis. mBio 14, e0082223 10.1128/mbio.00822-23 37535401 PMC10470552

[73] Bradley, J., Stone, W., Da, D.F., Morlais, I., Dicko, A., Cohuet, A. et al. (2018) Predicting the likelihood and intensity of mosquito infection from sex specific *Plasmodium falciparum* gametocyte density. Elife 7, e34463 10.7554/eLife.34463 29848446 PMC6013255

[74] Arrighi, R.B., Lycett, G., Mahairaki, V., Siden-Kiamos, I. and Louis, C. (2005) Laminin and the malaria parasite's journey through the mosquito midgut. J. Exp. Biol. 208, 2497–2502 10.1242/jeb.01664 15961736

[75] Saeed, S., Tremp, A.Z. and Dessens, J.T. (2023) *Plasmodium berghei* oocysts possess fatty acid synthesis and scavenging routes. Sci. Rep. 13, 12700 10.1038/s41598-023-39708-z 37543672 PMC10404217

[76] Stanway, R.R., Bushell, E., Chiappino-Pepe, A., Roques, M., Sanderson, T., Franke-Fayard, B. et al. (2019) Genome-scale identification of essential metabolic processes for targeting the Plasmodium liver stage. Cell 179, 1112–1128.e1126 10.1016/j.cell.2019.10.030 31730853 PMC6904910

[77] Smith, R.C. and Barillas-Mury, C. (2016) Plasmodium oocysts: overlooked targets of mosquito immunity. Trends Parasitol. 32, 979–990 10.1016/j.pt.2016.08.012 27639778

[78] Tremp, A.Z., Saeed, S., Sharma, V., Lasonder, E. and Dessens, J.T. (2020) *Plasmodium berghei* LAPs form an extended protein complex that facilitates crystalloid targeting and biogenesis. J. Proteomics 227, 103925 10.1016/j.jprot.2020.103925 32736136 PMC7487766

[79] Saeed, S., Tremp, A.Z. and Dessens, J.T. (2018) The Plasmodium LAP complex affects crystalloid biogenesis and oocyst cell division. Int. J. Parasitol. 48, 1073–1078 10.1016/j.ijpara.2018.09.002 30367865 PMC6284103

[80] Ukegbu, C.V., Christophides, G.K. and Vlachou, D. (2021) Identification of three novel Plasmodium factors involved in ookinete to oocyst developmental transition. Front. Cell. Infect. Microbiol. 11, 634273 10.3389/fcimb.2021.634273 33791240 PMC8005625

[81] Bailey, A.J., Ukegbu, C.V., Giorgalli, M., Besson, T.R.B., Christophides, G.K. and Vlachou, D. (2023) Intracellular Plasmodium aquaporin 2 is required for sporozoite production in the mosquito vector and malaria transmission. bioRxiv 10.1101/2023.03.15.532816 PMC10622946 37883438

[82] Dessens, J.T., Tremp, A.Z. and Saeed, S. (2021) Crystalloids: fascinating parasite organelles essential for malaria transmission. Trends Parasitol. 37, 581–584 10.1016/j.pt.2021.04.002 33941493

[83] Saeed, S., Tremp, A.Z. and Dessens, J.T. (2015) Biogenesis of the crystalloid organelle in Plasmodium involves microtubule-dependent vesicle transport and assembly. Int. J. Parasitol. 45, 537–547 10.1016/j.ijpara.2015.03.002 25900212 PMC4459735

[84] Ecker, A., Bushell, E.S., Tewari, R. and Sinden, R.E. (2008) Reverse genetics screen identifies six proteins important for malaria development in the mosquito. Mol. Microbiol. 70, 209–220 10.1111/j.1365-2958.2008.06407.x 18761621 PMC2658712

[85] Ukegbu, C.V., Gomes, A.R., Giorgalli, M., Campos, M., Bailey, A.J., Besson, T.R.B. et al. (2023) Identification of genes required for Plasmodium gametocyte-to-sporozoite development in the mosquito vector. Cell Host Microbe 31, 1539–1551.e6 10.1016/j.chom.2023.08.010 37708854 PMC7618085

[86] Merrick, C.J. (2021) Hypnozoites in Plasmodium: do parasites parallel plants? Trends Parasitol. 37, 273–282 10.1016/j.pt.2020.11.001 33257270

